# Dr. Nicholas Senn

**Published:** 1908-02

**Authors:** 


Dr. Nicholas Senn, of Chicago, died at his home in that city January 2, 1908, in his 64th year, of dilatation of the heart.

Dr. Senn was born in the village of Buchs, Canton of St. Gall, Switzerland, on October 31, 1844. When he was nine years old his parents brought him to this country and settled at Ashford, Wis., where he was brought up on a farm. After being graduated from the high school he taught school for three years, at the same time attending the lectures at the Chicago Medical College. He was graduated from this latter institution in 1868, winning the first prize for his thesis. He then spent a year and a half as house physician in the Cook County Hospital, and from 1869 to 1874 practised his profession in Fond du Lac. Then he moved to Milwaukee, where he remained nearly twenty years. He was appointed surgeon general of the Wisconsin National

Guard, and organised the Association of Military Surgeons of the United States, becoming its first president.

In 1884 Dr. Senn was appointed professor of surgery in the College of Physicians and Surgeons, in Chicago, and in 1887 was elected professor of the principles of surgery and surgical pathology in Rush Medical College, and in 1890 professor of practical and clinical surgery. After 1891 he made his home in Chicago. He was surgeon general of the Illinois National Guard on the staffs of several successive Governors.

When the Spanish-American war broke out Dr. Senn offered his services to the government. He abandoned his practice for active work in the field at Chattanooga, Tenn.; at Santiago, Cuba; at Ponce, P. R., and at Montauk Point, Long Island. Many of the successful steps taken to maintain the health of the soldiers were taken at his instance. He was made chief surgeon of the 6th Army Corps, with the rank of lieutenant colonel of volunteers, and chief of the operating staff with the army in the field.

In September, 1898, after peace had been assured Dr. Senn resumed his professional work in Chicago. He was a delegate to the International Medical Congress at Berlin in 1890, at Moscow in 1897 and at Madrid in 1903. Besides his connection with Rush Medical College, he was professor of surgery at the Chicago Polyclinic, attending surgeon of the Presbyterian Hospital and surgeon in charge of St. Joseph's Hospital.

Dr. Senn wrote a number of books of great value to the medical world, among them "Surgical Bacteriology," "Principles of Surgery," "Experimental Surgery," "Practical Surgery for the General Practitioner," "Tuberculosis of the Bones and Joints," "Surgical Notes on the Spanish-American War," "The Pathology and Treatment of Tumors," and "Around the World via India—A Medical Tour." His private library was regarded as perhaps the finest of its kind in the country. In 1869 he married Miss Aurelia Muehlhauser, of La Crosse, Wis., who survives him.

